# Properties of Alkali Activated Lightweight Aggregate Generated from Sidoarjo Volcanic Mud (Lusi), Fly Ash, and Municipal Solid Waste Incineration Bottom Ash

**DOI:** 10.3390/ma13112528

**Published:** 2020-06-02

**Authors:** Puput Risdanareni, Yury Villagran, Katrin Schollbach, Jianyun Wang, Nele De Belie

**Affiliations:** 1 Magnel-Vandepitte Laboratory for Structural Engineering and Building Materials, Ghent University, Tech Lane Ghent Science Park, Campus A, Technologiepark Zwijnaarde 60, B-9052 Ghent, Belgium; puput.risdanareni@ugent.be (P.R.); Yury.VillagranZaccardi@UGent.be (Y.V.); 2 Department of Civil Engineering, Faculty of Engineering, State University of Malang, Semarang Street 5, Malang 65145, Indonesia; 3 Laboratory for Multidisciplinary Training in Technological Research, National Scientific and Technical Research Council, 52 entre 121 y 122 s/n, La Plata 1900, Argentina; 4 Department of the Built Environment, Eindhoven University of Technology, 5612AP Eindhoven, The Netherlands; K.Schollbach@tue.nl; 5 Department of Civil Engineering, Xi’an Jiaotong University, Yanxiang Road 99, Xi’an 710054, China; jianyun.wang@xjtu.edu.cn

**Keywords:** lightweight aggregate, fly ash, bottom ash, Sidoarjo mud, mortar, fine aggregate replacement

## Abstract

Production of artificial lightweight aggregate (LWA) from industrial by-products or abundant volcanic mud is a promising solution to prevent damaging the environment due to the mining of natural aggregate. However, improvements are still needed in order to control the high water absorption of LWA and strength reduction in resulting concrete or mortar. Hence in this research, fly ash, municipal solid waste incineration bottom ash (MSWI BA), and Sidoarjo volcanic mud (Lusi) were employed as a precursor and activated using NaOH 6 M and Na_2_SiO_3_ in producing LWA. The influence of the type of the precursors on the physical properties of resulting LWA was investigated. The effect of replacing natural fine aggregate with the resulting LWA on the compressive strength and volume density of mortar was also determined. Finer particles, a high amount of amorphous phase, and low loss on ignition (LOI) of the raw material improved the properties of resulting LWA. Mortar compressive strength was decreased by 6% when replacing 16% by volume of natural fine aggregate with fly ash based LWA. Compared to the expanded clay LWA, the properties of alternative LWAs in this study were slightly, but not significantly, inferior. Alternative LWA becomes attractive when considering that expanded clay LWA requires more energy during the sintering process.

## 1. Introduction

The global demand for aggregates is increasing along with increasing concrete demand. In 2015, the demand reached 48.3 billion tons per year, with an expected annual increase of 5.2% [1]. With the increasing demand for aggregates, especially in the concrete industry, the availability of natural aggregate will decrease as this material is non-renewable and continued mining will lead to environmental damage. Hence, developing a new artificial aggregate from industrial waste and abundant volcanic material could be a promising solution.

Sidoarjo volcanic mud (Lusi) is an indigenous Indonesian material resulting from a volcanic eruption at Sidoarjo city, East Java, Indonesia in May 2006. Until now, as much as 50 million m^3^ of this mud has been placed in a giant embankment with an area of 640 ha. This eruption caused a massive loss of habitable land at Sidoarjo and was recognized as a national disaster in Indonesia [2]. Therefore, the Indonesian government has been looking for a suitable solution to utilize this abundant material. Since 2007, several attempts have been made by Indonesian researchers to utilize Lusi as a construction material (e.g., as a cement substitute or precursor for geopolymer material due to its high silica and alumina content [3,4,5]). It is reported that cement production in Indonesia reached 63 million tons in 2019 [6]. As the maximum replacement rate of cement by Lusi was 40%, around 25 million tons of Lusi could be used in this application [5]. On the other hand, beneficiating Lusi for use in lightweight aggregate (LWA) would be an alternative option. Indeed, the global production of LWA is low compared to cement production and only reached 1% of the total aggregate demand, which is less than 1 billion tons per year [1]. However, the global demand of LWA will grow quite rapidly along with the increasing demand of lightweight structural concrete, which is expected to have an annual increase of 7.1% [1].

Other abundant raw material sources are coal combustion fly ash and municipal solid waste incineration bottom ash. In 2015, it was reported that the global production of coal combustion products (CCPs) such as fly ash was around 780 million metric tons, which is expected to increase 1.6% each year [7]. Although more than 50% of those CCP was successfully utilized, there is still a huge amount of fly ash available. On the other hand, around 130 million tons of municipal solid waste (MSW) are generated each year around the world [8]. Most of the non-hazardous fraction of this MSW is co-incinerated, with the heat produced being converted into electrical energy. The largest portion of incineration plant by-products is bottom ash. This is mostly landfilled, which is not a good option as it can result in high groundwater pollution due to its heavy metal content [9]. It is also reported that treated Municipal Solid Waste Incineration (MSWI) bottom ash, which is rich in silica and alumina, could be a potential alternative cement replacement in concrete production [8]. Combining fly ash and MSWI bottom ash as a precursor in geopolymer production was also proven to improve the properties of the resulting geopolymer concrete [10]. Hence, Lusi, fly ash, and MSWI bottom ash are interesting candidates to mass produce LWA.

Transforming fly ash (FA), municipal solid waste incineration bottom ash (MSWI BA), and Lusi into artificial lightweight aggregate is not a new technique. However, most of the research has been focused on sintering and cold bonding with cement [11,12,13,14]. There are limited studies on applying alkali activation to produce LWA, even though this binding system can improve the properties of resulting LWA [15,16].

In an alkali activated binder system, the type of alkali activator [17], the concentration [15,18,19], the curing regime [20,21], and the type of precursors [14,22,23] were reported as important parameters for the properties of the resulting geopolymer products such as paste, mortar, concrete, and LWA. Based on literature, the dissolution rate of material, which is rich in silica and alumina, is higher in NaOH than in KOH solution [17]. As Lusi, fly ash, and bottom ash are known for their high silica and alumina content, NaOH was chosen as the alkali activator in this study. In the literature, the ideal concentration of NaOH for producing a geopolymer paste is reported to be in the range of 8–12 M [18,20,24]. On the other hand, Gorhan et al. showed that the optimum concentration of NaOH for better properties of fly ash geopolymer paste is 6 M [15]. The weight ratio between liquid/solid in the current study was lower compared to geopolymer paste, as in LWA production, the mixture should be a bit drier so that the granule can roll and does not stick to the pan granulator. A lower concentration of NaOH was also required to avoid workability issues during the pelletization process. Hence, in this study, NaOH with a concentration of 6 M was chosen to produce LWA.

In summary, the objective of this research was to investigate the effect of Lusi, fly ash, and MSWI bottom ash as binders on the properties of geopolymer-based LWA. Several properties were investigated such as water absorption, density, particle size distribution, heat evolution of the precursor during reaction with the activator, pore size distribution, crushing resistance, and mineralogical composition. The LWA was also applied in mortar as a natural fine aggregate substitution. The compressive strength and the bulk density of mortar containing LWA were determined. In the end, the properties of LWA and mortar containing LWA were compared to commercial expanded clay LWA in order to demonstrate the possibility of applying this novel LWA in the construction industry.

## 2. Materials and Methods

### 2.1. Materials

Fly ash, MSWI bottom ash, and Sidoarjo mud (Lusi) were utilized as a binder. The fly ash (FA) was obtained from a coal fired power plant in the Netherlands and can be classified as class F based on its chemical composition. MSWI BA, with a fraction of 2–6 mm, was obtained from a municipal solid waste incinerator (Indaver, Mechelen, Belgium), dried for 24 h at 105 °C, and ground into powder using a ball mill. Lusi, an indigenous volcanic mud from Indonesia, was oven-dried for 24 h at 105 °C, ground into powder, and then calcined at 800 °C for 6 h to activate the silica and alumina oxide in the Lusi.

The powder form of sodium hydroxide (NaOH) with a purity of 98% (VWR International Belgium Bvba, Leuven, Belgium) and liquid form of sodium silicate (Na_2_SiO_3_) (VWR International Belgium Bvba, Leuven, Belgium), which contains 26.3% silica, 7.9% sodium oxide, and 65.8% water, were used as an alkali activator in this research. NaOH solution with a concentration of 6 M was obtained by dissolving 240 g of NaOH powder in 1 L of distilled water one day before use. Later on, Na_2_SiO_3_ was mixed with the NaOH 6 M solution with a weight ratio of 1.5. Finally, the mixed alkali activator was left at room temperature for one day prior to use.

Commercial expanded clay (EC) based LWA (Argex NV, Antwerp, Belgium) was used as the reference. This LWA was produced by sintering natural clay at a temperature of 1100 °C in a rotating kiln. The nominal size of EC LWA is 0/4.

Portland cement type I 52.5 (Holcim, Mons, Belgium) was used as a binder in mortar application while standard river sand with the fraction of 0/4 was used as the fine aggregate.

### 2.2. Experimental Methods

#### 2.2.1. Pelletizing Procedure

The values for the parameters in LWA manufacturing such as the diameter, the slope angle, and the speed of the rotating pan, were chosen based on previous research by Baykal [25]. The pan granulator (EIRICH, TR04, Hardheim, Germany) had a diameter of 80 cm, the slope of the pan was set to 48°, and the speed of 60 rpm was applied.

**Figure 1:**
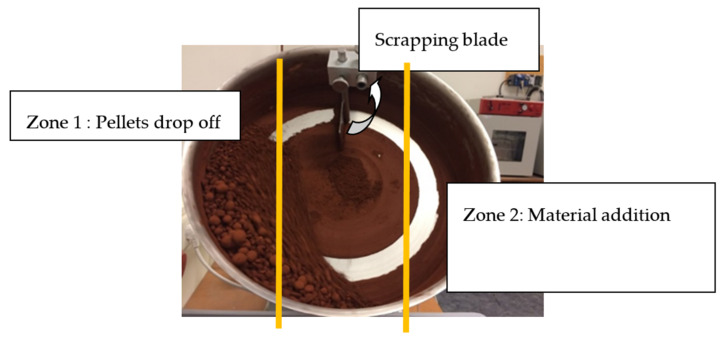
Illustration of the granulation process.

Around 5 kg of dry binder powder was added into the rotating pan, followed by spraying alkali activator liquid continuously. After approximately 20 min, the wet pellets that formed fell out of the pan automatically (Figure 1). The wet pellets were dried for 24 h in a curing room that had a temperature of 20 °C and a relative humidity of 95 ± 5%. These were then stored in a sealed plastic bag until the testing date.

The amount of alkali activator liquid that was added to the mixture was varied, depending on the type of raw material used as the binder. The amount of liquid needed was determined experimentally for each raw material based on its water absorption and the pellet consistency. The mixture proportion of each LWA is presented in Table 1, while the illustration of the granulation process using a pan granulator is displayed in Figure 1.

#### 2.2.2. Characterization of the Samples

A laser diffraction apparatus (Malvern Mastersizer 2000, Malvern, UK) was used to measure the particle size distribution of all the binder powders after dispersion in isopropanol (VWR International Bvba, Leuven, Belgium). The absorption index of all raw materials was 0.1, while the refractive indexes for fly ash, MSWI bottom ash, and Lusi were 1.67, 1.70, and 1.57, respectively. The obscuration level was maintained between 12 and 15. During the measurements, the dispersant and the sample were stirred with the speed of 800 rpm. The d_50_ values were obtained as the average value of six measurements.

The particle size distribution of LWA was determined using standard sieves according to NBN EN 12620. A sieve column with standardized sieves with sieve openings of 63, 45, 40, 31.5, 22.4, 20, 16, 14, 12.5, 10, 8, 6.3, 4, 2, 1, 0.5, 0.25, 0.125, and 0.063 mm was used. The cumulative mass percentage of LWA passing through each sieve was measured and then plotted in the particle size distribution graph.

The chemical composition of the binders was determined by X-ray fluorescence (XRF) measurements. These tests were carried out using Rigaku NeXCG equipment on a loose powder sample (Tokyo, Japan). The excitation is provided by a close-coupled 50 KV/50 W Pd-anode end-window X-ray tube. The tube was fitted with a shutter to maintain its stability and durability. Finally, the spectra from the sample were recorded by a silicon drift detector (SDD). The chemical compositions of the raw materials were obtained as the average of three measurements.

Heat evolution of fresh LWA was monitored using an isothermal calorimeter (TA instrument, New Castle, DE, USA). All raw materials were put in the climate room with a temperature of 20 ± 2 °C one day before use. Around 10–15 g of precursor was mixed with alkali activator in a glass ampoule. The weight ratio between the liquid and solid of FA 6 M, Lusi 6 M, and MSWI BA 6 M was 0.25, 0.51 and 0.26, respectively, like for the LWA as shown in Table 1. The ampoules filled with paste samples were then placed into a TAM air isothermal calorimeter and measured at 20 °C for 7 d.

The apparent density (*ρ_a_*), the oven dried density (*ρ_OD_*), the water saturated surface-dry density (*ρ_SSD_*), and water absorption over 24 h (*WA*_24_) were determined by following the NBN EN 1097–6 (2013) standard. Around 500 g of dried LWA was immersed in water in a calibrated flask. After that, the flask was stacked in a water bath for 24 h and was weighed (*M*_2_). Later, the LWA was dried until reaching the saturated surface dry condition and were weighed (*M*_1_). The SSD LWA was then dried in an oven with a temperature of 110 ± 5 °C until reaching a constant mass, and was weighed again (*M*_4_). Meanwhile, the flask was refilled with water to the same volume as before and weighed (*M*_3_). Finally, the apparent density (*ρ_a_*), the oven dried density (*ρ_OD_*), the water saturated surface-dry density (*ρ_SSD_*), and water absorption over 24 h (*WA*_24_) were calculated using Equations (1)–(4).

(1) $$\rho_{a}=\rho_{w}\frac{M_{4}}{M_{4}-\left(M_{2}-M_{3}\right)}$$

(2) $$\rho_{od}=\rho_{w}\frac{M_{4}}{M_{1}-\left(M_{2}-M_{3}\right)}$$

(3) $$\rho_{SSD}=\rho_{w}\frac{M_{1}}{M_{1}-\left(M_{2}-M_{3}\right)}$$

(4) $$WA_{24}=\frac{100\times \left(M_{1}-M_{4}\right)}{M_{4}}$$

where *ρ_w_* is the density of water measured at 20 °C, which is 998 kg/m^3^. The values reported for density and water absorption are the average value from three replicates, while the standard deviation on individual values was also calculated.

Mercury intrusion porosimetry (MIP) was performed to analyze the pore size distribution of LWA. In order to dry the samples without damaging the microstructure, freeze-drying was applied [26]. LWA was immersed in liquid nitrogen for 5 min and put in the freeze dryer vacuum chamber with a pressure of 0.1 Pa for two weeks until constant mass was achieved. Afterward, around 1.7 g LWA was filled into the dilatometer and was tested in a Pascal 140 and Pascal 440 machine. The pressure were then being increased with the speed of 5–17 MPa/min. Finally, the pressure needed for mercury to penetrate into the pores of the sample is expressed in Equation (5) [27].

(5) p=−4γcos(θ)/d

where *P* is the pressure needed; *γ* is the surface energy of mercury (483 mN/m); *θ* is the contact angle (142°); and d is the pore diameter of the sample. In order to avoid damaging the microstructure of the sample, the maximum pressure was limited to 200 MPa.

The mechanical properties of LWA were assessed by performing a crushing resistance test according to NBN B11-205. A cylindrical steel container with a diameter of 75 mm was filled with oven-dried LWA. After that, a no-friction plunger was put on the top of the container and forced down with a speed of 0.42 kN/s until 100 kN was reached. This load was maintained for 2 min. Later, the crushed aggregates with a diameter larger than 2 mm were separated by means of sieving. Finally, the crushing resistance value of the aggregate was the mass ratio between the crushed aggregate with a fraction bigger than 2 mm over the initial weight of the sample.

The mineralogy of LWA was assessed by X-ray diffraction (XRD). For the quantitative XRD (QXRD), the Rietveld method with zinc oxide (ZnO) as an internal standard was used. First, the LWA sample was pre-ground, then mixed with ZnO (10% wt). The pre-ground LWA and ZnO mix was ground all together in a McCrone mill (Retsch) until a size of about 50 microns was reached. A D4 (Bruker) with a Co-tube (Kα_1_ 1.7901 Å, Kα_2_ 1.7929 Å) and Lynx eye detector were used for the measurement. The settings were fixed to divergence slits (0.5°), 0.04 rad Soller slits, and a step rate of 0.02° 2θ/s.

#### 2.2.3. Application of LWA in Mortar

In this research, sand with a fraction of 2/4 was substituted by volume with LWA of the same fraction to produce mortars. In order to avoid the detrimental effects of the high water adsorption of LWA during the mortar production, LWA was treated to obtain the saturated surface dry condition before it was added into the mortar mixture. The mix design of each mortar including the amount of LWA in oven-dried condition and the absorbed water needed to achieve the SSD condition is presented in Table 2.

The mortar was molded into prisms with dimensions of 40 × 40 × 160 mm. After demolding, the mortar prisms were cured until the age of 28 d in a room with a temperature of 20 °C and relative humidity of 95 ± 5%. The mechanical properties of the mortars were assessed by measuring the flexural and compressive strength in accordance with EN NBN 196. First, the dimension and the mass of the prism were measured. After that, the prism was tested in an apparatus for three-point bending (Walter + Bai 250/15). The load was increased smoothly with the rate of 2400 ± 200 N/s. Later, the halves of the broken prisms from the flexural test were subjected to a uniformly distributed load to measure compressive strength.

## 3. Results and Discussion

### 3.1. Properties of Raw Materials

Table 3 shows the chemical composition of the binders determined by X-ray Fluorescence (XRF). It can be seen that MSWI BA had the lowest amount of SiO_2_, Al_2_O_3_, and Fe_2_O_3_, but had the highest amount of CaO. FA and Lusi had similar amounts of SiO_2_, Al_2_O_3_, Fe_2_O_3_, and CaO. It was also shown that Lusi had the highest loss on ignition (LOI), followed by FA and MSWI BA. The specific density of Lusi and MSWI BA were similar, while FA had the lowest specific density.

The mineralogy of raw materials shows that Lusi had the lowest amorphous content, followed by MSWI BA and FA (Table 4). The amount of amorphous phase in MSWI BA was quite high, but this value was similar to the value reported earlier for fresh MSWI BA obtained from the Belgian incinerator, being 73% [28]. The major crystalline phases were quartz, mullite, and calcite. Lusi had the highest quartz content followed by MSWI BA and FA, while calcite was only detected in MSWI BA and Lusi.

**Figure 2:**
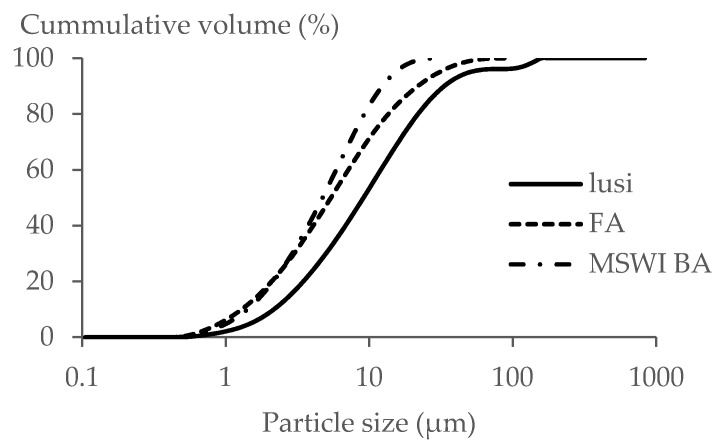
Particle size distribution.

Figure 2 displays the particle size distribution (PSD) of all the powders (FA, Lusi, and MSWI BA, (Indaver, Mechelen, Belgium)). It can be seen that FA and MSWI BA had a very similar size distribution, while the PSD curve of Lusi indicates that it contained a higher content of large particles (Figure 2). The d_50_ of Lusi, FA, and MSWI BA was 10.5 µm, 6.2 µm, and 5.4 µm, respectively (Figure 2). The surface area of Lusi, FA, and MSWI BA was 0.51 m^2^/g, 0.83 m^2^/g, and 0.65 m^2^/g, respectively.

**Figure 3:**
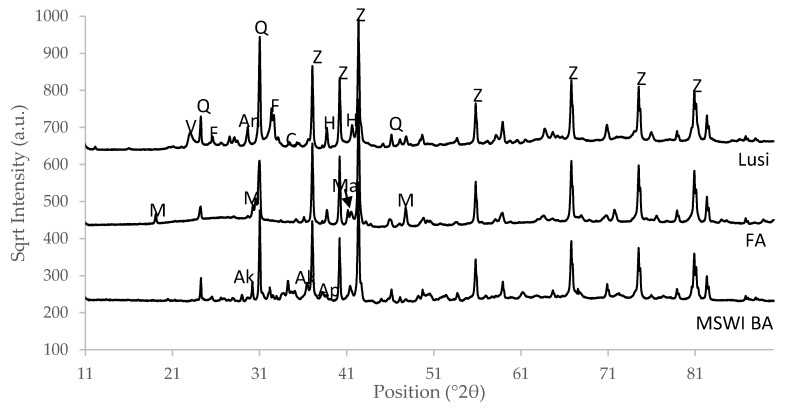
X-ray diffraction (XRD) pattern of raw materials, showing the square root of intensity versus the 2θ position. Q: Quartz, F: Feldspar, Z: ZnO, Ak: Akermanite, A: Anhydrite, V: Vermiculite, C: Calcite, M: Mullite, Ap: Apatite, Ma: Magnetite.

The XRD pattern of the raw materials showed that FA had a less crystalline phase compared to Lusi and MSWI BA (Figure 3). A small broad hump in the region of 21–31° was also clearly observed in FA, indicated its high amorphous phase content. Quartz could be detected in all of the raw materials, while mullite was only detected in FA and Lusi.

### 3.2. Heat Evolution

**Figure 4:**
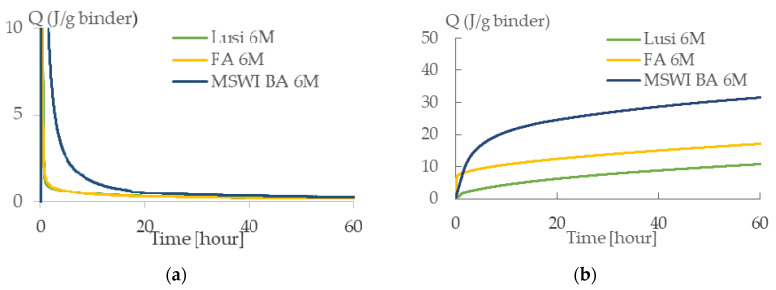
(**a**) Heat evolution of LWA. (**b**) Heat cumulative of LWA.

Based on the heat evolution shown in Figure 4, it can be seen that all of the LWA had low reactivity, indicated by the absence of a significant heat flow after the initial mixing. The cumulative heat of all LWA was also quite low, with MSWI BA 6 M releasing the highest heat followed by FA 6 M and Lusi 6 M. The total released heat of MSWI BA 6 M, FA 6 M, and Lusi 6 M amounted to 40.47 J/g, 25.97 J/g, and 19.03 J/g, respectively. This result was also influenced by the different average particle size of the three materials. The total heat released was inversely proportional to the particle size of the raw material.

The absence of a heat flow following the initial heat release that occurs directly after mixing was also observed while activating fly ash, slag, and metakaolin with NaOH at ambient temperatures [29,30,31]. As the NaOH concentration increases, more energy (higher temperature) is required to initiate and forward the reaction [29]. Therefore, it makes sense that samples cured at room temperature had a single peak pattern. There was not enough energy to initiate further geopolymerization and the alkali solution produced a slow reaction with fly ash and caused difficulties in observing all the possible peaks.

### 3.3. Density and Water Absorption

Based on data presented in Table 5, it can be seen that the pellets MSWI BA 6 M had the highest apparent density, saturated surface dry (SSD) density, and oven-dried (OD) density. Lusi 6 M had the lowest OD density, but also one of the highest apparent densities. All the LWA had a water absorption of more than 20%. Lusi 6 M had the highest water absorption and porosity values, followed by MSWI BA 6 M and FA 6 M.

In general, water absorption and porosity of LWA had inverse relationships with density [11]. In contrast, in this research, it was quite difficult to draw this conclusion. The apparent density, the oven-dried (OD) density, and the SSD density of all of the LWAs showed no single trend. The results for water absorption and density for FA 6 M and MSWI BA 6 M are not fully consistent. Whereas MSWI BA 6 M had the highest OD density, it would be expected that it also had the lowest water absorption. However, the lowest water absorption was from FA 6 M, with a difference of less than 2% compared to the water absorption of MSWI BA 6 M. The main explanation for this inconsistency seems to be the microstructure of the different LWAs, which will be further discussed in this paper.

Compared to bottom ash geopolymeric LWA, produced with 8 M concentration NaOH and Ca(OH)_2_ admixture, the water absorption of the LWA in this study was higher [32]. This makes sense as the increased NaOH concentration and the addition of calcium enhanced the geopolymer reaction. An insufficient amount of calcium and alkali activator reduces the reactivity of the geopolymer and increases the amount of unreacted phase in the resulting LWA. Moreover, unreacted phases in LWA cause an increase in the open porosity. The denser structure of FA 6 M and MSWI BA 6 M, made with binders with high calcium contents, supports this hypothesis. Furthermore, Gesoglu et al. explained that the particle size of LWA has a proportional relationship with its strength and density [12]. Thus, another possible explanation for the high water absorption of LWAs in the present research is its smaller particle size fraction compared to the LWA in the literature.

### 3.4. Particle Size Distribution

**Figure 5:**
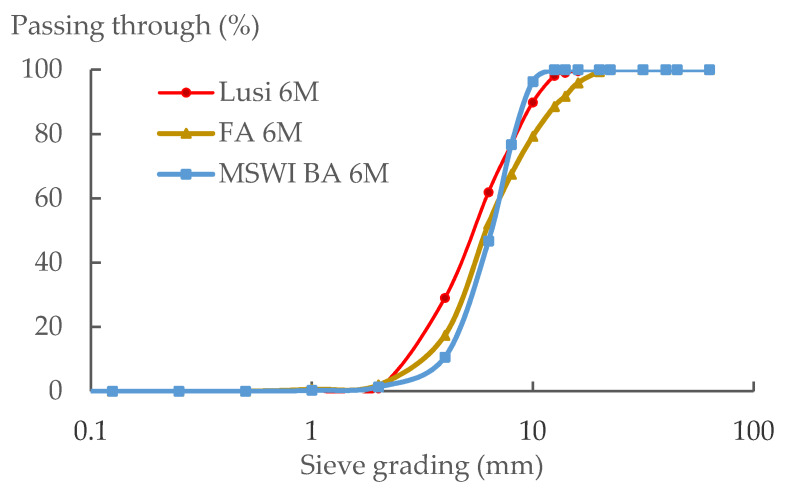
Particle size distribution of the LWA.

Figure 5 shows that all specimens had a very small content of fine particles of less than 2 mm. Lusi 6 M had the least steep curve, and a higher fraction of fine particles compared to the other LWAs. According to NBN EN 12620, the Lusi 6 M and MSWI BA 6 M LWA meet the requirements for the 2/8 fraction, while FA 6 M has a fraction of 2/10. Thus, it can be noted that the types of raw materials did not affect the particle size distribution of LWA in any significant way. Based on the literature, it is mainly the setup of the pan granulator such as the slope degree, the speed, and the duration of pelletizing that affect the particle size of the resulting LWA [25].

### 3.5. Crushing Resistance Test

**Figure 6:**
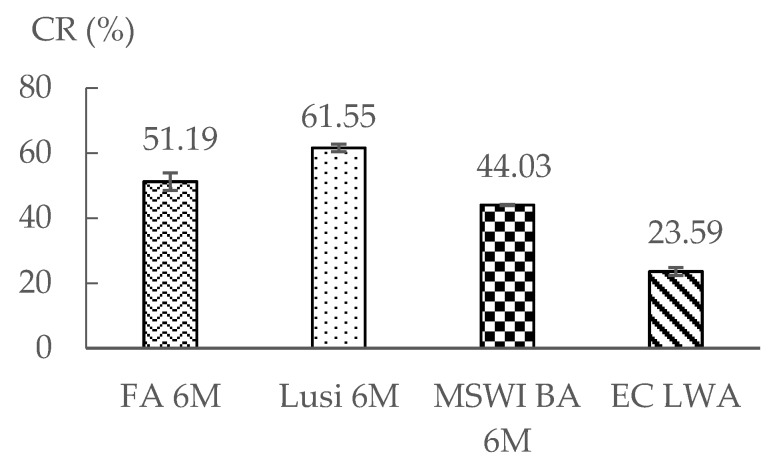
Crushing resistance (CR) value of LWA.

**Figure 7:**
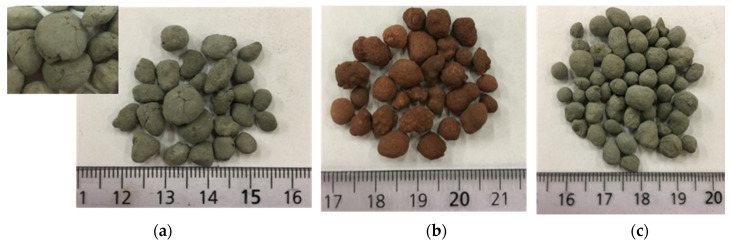
The appearance of (**a**) MSWI BA 6 M, (**b**) Lusi 6 M, (**c**) FA 6 M.

Based on Figure 6, Lusi 6 M had the highest crushing resistance (CR), followed by FA 6 M and MSWI BA 6 M. This result is unexpected, as Lusi 6 M had the highest water absorption among the three LWAs. In general, the CR value of aggregates has an inverse relationship with its water absorption [7,33,34]. MSWI BA 6 M showed low strength, even though it had high reactivity. This is likely due to the appearance of micro cracks on the surface of the aggregates (Figure 7). This could occur due to the metallic Al in the bottom ash, which reacts at a high pH and generates hydrogen gas that leads to expansion. Based on previous research, the aluminum content in the MSWI BA was 0.80% [35]. Furthermore, the result of the MIP test also confirmed that MSWI BA 6 M contains a high number of macro-pores compared to the other LWAs, because, even without the influence of metallic Al, the porosity of the MSWI BA fines tended to be very high [36]. The CR result was also not well correlated with the calorimetry test (Figure 3), where MSWI BA 6 M, which showed the highest cumulative heat, did not possess the highest CR. Previous research reported that NaOH activated fly ash is more temperature dependent and observing the reactivity of this paste in a 20 °C environment will not provide enough information to understand the whole kinetics of the reaction that occurred [37].

It was observed during the test that all types of LWA were agglomerated and stuck to each other, forming bigger granules once the load for the crushing test had been applied. Thus, it was quite difficult to accurately determine the crushed part of the LWA with the method applied in this research.

Due to the different standard that was used, the CR value of the LWA in this research could only be compared with the limited results on LWA in the literature. Compared to crushed LWA generated from bottom ash and from limestone in the literature, the CR value of LWA in this research was quite similar to limestone aggregate [38]. Furthermore, the fact that the LWAs produced in this study had a similar CR value compared to those LWAs in the literature is quite promising, considering that the former had a much lower density and higher water absorption than the latter.

### 3.6. Porosity

**Figure 8:**
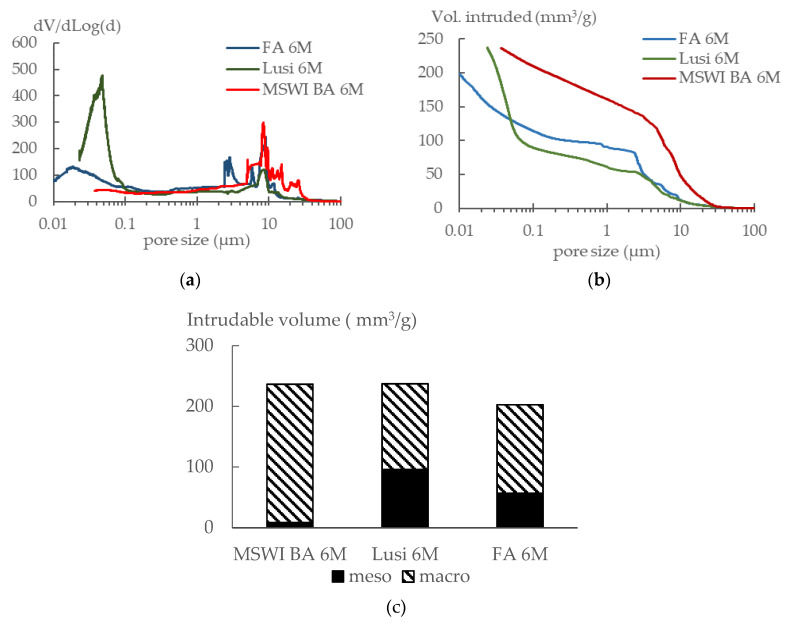
(**a**) Pore size distribution of LWA. (**b**) Cumulative intruded volume of LWA. (**c**) Types of pores.

Figure 8a,b shows the pore size distribution and cumulative intruded volume, respectively, of the LWAs determined by mercury intrusion porosimetry (MIP). Figure 8a shows that Lusi 6 M and FA 6 M had two major peak regions. The first region is located between 0.1–1 µm, while the second peak region is located between 3–10 µm. In contrast, MSWI BA 6 M only showed one major peak region, which is located between 3–10 µm. As a consequence, two threshold pore diameters were observed in Figure 8b for FA 6 M and Lusi 6 M, while only one was observed for MSWI BA 6 M. The threshold pore diameters (d_th_) were 18 µm for MSWI BA 6 M, 0.06 µm and 6 µm for Lusi 6 M, and 0.1 µm and 6 µm for FA 6 M. In addition, Figure 8b also shows that MSWI BA 6 M and Lusi 6 M had almost the same intruded volume of 238 mm^3^/g, while FA 6 M had a lower intruded volume of 200 mm^3^/g.

The porosity in the LWA samples can be categorized into two groups: MSWI based on their size range, mesopores (2–50 nm), and macropores (larger than 50 nm) [39]. As shown in Figure 8c, MSWI BA 6 M had the lowest volume of mesopores followed by FA 6 M and Lusi 6 M. The relative volumes of mesopores in MSWI BA 6 M, FA 6 M, and Lusi 6 M were, respectively, 3.48%, 28.03%, and 40.31% of the total porosity.

Tziviloglou et al. found multiple peaks for the pore size distribution curve of expanded clay LWA Liapor [40]. Other studies on the characterization of the pore structure of LWA also found multiple peaks in their pore size distribution curves [41]. This condition presumably occurs due to the fracture of the pore walls when pressure is applied in order to force mercury into the pores. Compared to sintered fly ash aggregate, which used bentonite and glass powder as a binder, a larger critical pore size diameter was obtained in this research. This seems logical as during the sintering process, the voids are better closed by the binders.

The appearance of a sharp peak in the mesopore range for FA 6 M and Lusi 6 M indicates the presence of ink-bottle pores. Indeed, this is a common criticism made to the MIP technique. Whereas it is intended to measure the connected open pore volume, misleading information regarding the pore size distribution may be obtained due to the presence of ink-bottle pores [41]. In this case, when large pores are connected by small channels, high pressure is needed to force the mercury through the small channel into the large pore, so a large volume of small pores shows up in the pore size distribution instead of the large pores that are actually present.

### 3.7. Mineralogy

**Figure 9:**
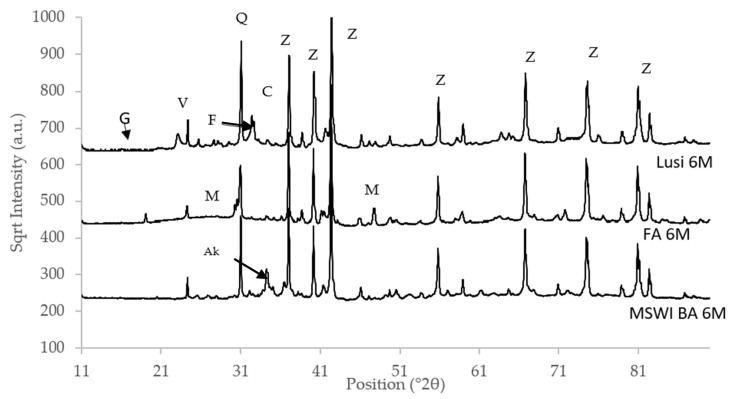
XRD pattern of the resulting LWA, showing the square root of intensity versus the 2θ position. Q: Quartz, F: Feldspar, Z: ZnO, Ak: Akermanite, V: Vermiculite, C: Calcite, M: Mullite, G: Gaylusite.

In all types of LWA, the number of crystalline phases such as quartz and mullite were decreased or not detected anymore after geopolymerization (Table 6). These minerals are not typically reactive in a geopolymer, so their decrease is due to the amount of alkali activator added into the mixture, which was quite high (around 25% for FA and MSWI BA LWA and 51% for Lusi LWA), which effectively dilutes the mineral content. Based on the XRD pattern in Figure 9, the broad hump of the amorphous phase was detected in the region of 25–35°. The location of the broad hump in the resulting LWA had shifted, compared to the raw materials (Figure 3). The same shifted mechanism also occurred in the previous study as an indication of aluminosilicate gel formation [42].

The amorphous phase contents from the quantitative XRD correlated well with the compressive strength of mortars containing these LWAs. Reports in the literature indicate that the amorphous phase content in the alkali activated paste has a directly proportional relationship with its compressive strength [42,43]. The FA 6 M LWA had a high content of amorphous phase as well as a high mortar compressive strength. On its own, the Lusi 6 M LWA showed the highest increase in the amorphous phase content due to its high alkali activator content, but the contribution of the aggregate to the compressive strength of mortar was lower. Therefore, it seems difficult to conclude which parameter in the raw material caused the unexpected behavior in the properties of the resulting LWA. The difference in reactivity, microstructure of raw materials, or the combination of both parameters could be the reason for this irregular trend.

### 3.8. Volume Density and Compressive Strength of Mortar

**Figure 10:**
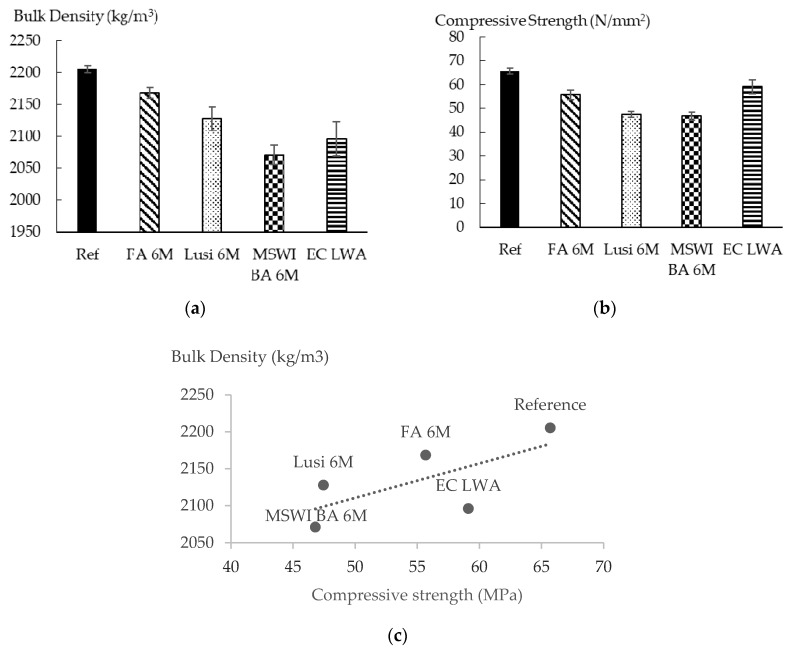
(**a**) Bulk density, (**b**) Compressive strength, (**c**) Correlation between compressive strength and bulk density of mortar containing LWA.

Figure 10a shows the bulk density of mortars made with the LWAs. The reference sample had the highest volume density, followed by FA 6 M, Lusi 6 M, and MSWI BA 6 M. As expected, replacing natural aggregate in a mortar matrix by LWA effectively reduces the volume density of mortar. Specifically, replacing 16% of natural aggregate can reduce the volume density up to 6%. The volume density of mortar incorporating LWA had no correlation with the density of the LWA. In terms of the OD density of LWA, Lusi 6 M was supposed to produce the lightest mortar, followed by FA 6 M and MSWI BA 6 M. However, MSWI BA 6 M generated the lightest mortar in correspondence with its highest macro-pore content among the LWAs.

As shown in Figure 10b, mortars containing FA 6 M LWA had the highest compressive strength followed by Lusi 6 M and MSWI BA 6 M. The strength reduction compared to the reference sample of FA 6 M, Lusi 6 M, and MSWI BA 6 M was 15.3 %, 27.8%, and 28.7%, respectively. This result agrees well with the volume density of mortars and the porosity values of LWAs. A proportional relationship can therefore be established between the compressive strength and the volume density of the produced mortars (Figure 10c).

In contrast, the compressive strength of the resulting mortar did not correlate well with the crushing strength of the constituting LWA. Lusi 6 M, which had the highest CR value, apparently did not deliver the highest compressive strength when it was applied in mortar. Aside from the difficulties with accurately determining the crushed part of LWA during the test, the reactivity of fly ash in FA 6 M LWA with the cement paste could also be the reason why the FA 6 M mortar had the highest compressive strength. In general, all the LWAs had a round shape covered with unreacted raw material powder. In mortar containing FA 6 M LWA, the unreacted fly ash in the aggregate could later react with the cement in the mortar mixture, creating a bond and close porosity, and increase the density. Lusi seems to have less reactivity compared to fly ash when applied into concrete or mortar. In the literature, a replacement level of 20% of cement by Lusi in concrete proved to decrease the strength activity index (SAI) up to 20% [5]. Previous research also indicated that fly ash LWA had pozzolanic reactivity when applied in mortar or concrete, especially at advanced ages [44]. The same reasoning might be used to explain why mortar containing FA 6 M also had a higher volume density, as the chemical reaction between the LWA and cement paste may have helped the mortar to become denser.

Compared to previous research by Ylinemi et al., who replaced 21% of fine aggregate in mortar by FA-based LWA, the compressive strength of mortar in the present study was 53% higher [33]. A comparison with the results reported by Gesoglu et al., who substituted 25 % of fine aggregate with a cement-bound fly ash LWA in mortar, shows that the decrease in strength in comparison with the reference mortar obtained in the present research was also much lower [10]. Whereas Gesoglu et al. obtained a reduction of 46% in the compressive strength at 28 days, the results the of FA 6 M mortar showed a reduction of only 15.3%. Even though this was partly due to a lower replacing percentage of 16%, the ratio of replacement percentage to strength reduction is much more convenient in the current results, indicating more proximity to the optimum replacement percentage.

In general, the LWAs produced in this study were comparable to other lightweight aggregates generated from similar waste products, and mortar produced with them led to better mechanical properties than those usually reported in the literature.

### 3.9. Comparison with Commercial LWA 

For practical validation, the LWAs produced in the present research were compared to a commercial aggregate generated from sintered expanded clay (EC). The fraction of EC LWA that was used in this research was 0/4 and its manufacturer states that this material is suitable for use in structural concrete. Table 5 shows that the oven-dried density of EC LWA was much lower than for all the LWAs produced in this research. Moreover, water absorption of EC LWA was 8% lower than the one of FA 6 M LWA, which showed the lowest water absorption among the LWAs that were produced. However, the CR value of this commercial LWA was 46% lower than the MSWI BA 6 M LWA, which had the lowest CR value among the produced LWAs (Figure 6). Surprisingly, with lower density and lower CR value, EC LWA demonstrated a better performance when applied into mortar. With a 16% replacing rate, mortar containing EC LWA only had a 9% strength decrease compared to the reference mortar (Figure 10). The volume density of mortar containing EC LWA was almost similar to the mortar containing MSWI BA 6 M, but it had better mechanical properties.

The sintering process is more efficient than geopolymerization in producing a convenient microstructure for LWA. This is the main explanation for the improved mechanical performance of EC LWA compared to the geopolymer LWA when applied in mortar. Previous reports on sintered fly ash based LWA revealed that the sintering process is effective in closing pores as the molten material forms a solid body [13,45]. Upon this processing, the volume of closed porosity increases at the expense of the open porosity that is blocked, with a consequential decrease in water absorption and improvement in the mechanical properties.

In summary, compared to EC LWA, the properties of LWA in this study are still acceptable. The properties of EC LWA were slightly better than those of the LWAs in this research. However, the sintering process is energetically expensive, as temperatures of up to 1200 °C are required to produce the full volume of aggregate. In this sense, a reduced demand of energy may compensate for the moderate performance of geopolymer LWA. This aspect should be considered in future research for appropriate comparison.

## 4. Conclusions

Based on the results of the experiment, the following conclusions could be drawn:

1. The types of raw materials used affected the water absorption and the density of the resulting LWAs. Fly ash and bottom ash contain more calcium and have a lower LOI than Lusi, so they delivered LWAs with a lower water absorption and denser structure compared to the LWA generated from Lusi.
2. The type of raw material had no significant effect on the particle size distribution of the resulting LWA, the fraction size of MSWI BA 6 M and Lusi 6 M LWA was 2/8 mm, while for the FA 6 M LWA, it was 2/10.
3. The MIP test results revealed that LWA generated from fly ash and Lusi had two threshold diameters (d_th_). FA 6 M LWA had a d_th_ of 0.1 µm and 6 µm while Lusi had a d_th_ of 0.06 µm and 6 µm. The largest d_th_ was measured for MSWI BA 6 M, which had a d_th_ of 18 µm. MSWI BA 6 M contained large amounts of macropores. The appearance of micro-cracks on the surface of the MSWI BA 6 M due to the metallic Al reaction could be the reason for the high number of measured macropores in the MSWI BA 6 M sample.
4. There was an unexpected result obtained from the crushing resistance test, where Lusi 6 M showed the highest crushing strength, followed by FA 6 M and MSWI BA 6 M. It seems that the crushing resistance test is not suitable in determining the strength of LWA that has a small particle size.
5. The compressive strength reduction compared to the equivalent mortar made with expanded clay LWA was in the range between 6% and 21% for mortar with FA 6 M LWA and mortar with MSWI BA 6 M LWA, respectively. LWAs produced in this study are comparable and have even better mechanical properties compared to other lightweight aggregates generated from the geopolymerization of similar waste products.
6. Despite the fact that the properties of mortar made with EC LWA were slightly better than that for the mortar made with LWAs in this research, the fact that more energy is required during the sintering process of EC LWA should also be a consideration.

## Figures and Tables

**Table 1:** Liquid/solid weight ratio of the lightweight aggregate (LWA).

Sample Codes	Liquid/Solid
FA 6M	0.25 ± 0.01
Lusi 6M	0.51 ± 0.01
MSWI BA 6M	0.26 ± 0.01

**Table 2:** Mix design of the mortar.

Type	Sand 2/4 (g)	LWA_OD_^1^ 2/4 (g)	Absorbed Water (g)	Sand 0/2 (g)	Cement (g)	Water (g)
Reference	220	0	0	1130	450	225
Lusi 6M	0	116	37	1196	450	225
FA 6M	0	122	32	1196	450	225
MSWI BA 6M	0	132	23	1195	450	225
EC LWA	0	82	17	1251	450	225

^1^ OD: oven dried.

**Table 3:** Chemical Composition of the binders determined via X-ray Fluorescence.

Composition (%)	MSWI BA	FA	Lusi
Cl	0.37	—	0.15
CaO	16.80	3.79	2.27
SiO_2_	48.40	57.40	52.90
Al_2_O_3_	10.10	26.17	22.20
Fe_2_O_3_	7.69	5.99	7.64
K_2_O	1.13	1.88	1.59
Na_2_O	6.43	—	—
MgO	2.98	1.43	2.52
CuO	0.25	0.02	0.01
ZnO	0.47	0.02	0.01
SO_3_	2.06	0.98	1.83
P_2_O_5_	1.91	0.88	—
TiO_2_	1.27	1.13	0.81
LOI	0.15	0.32	8.08
Specific Density (g/cm^3^)	2.61	1.99	2.75

**Table 4:** Quantitative X-ray Diffraction of municipal solid waste incineration bottom ash (MSWI BA), fly ash (FA), and Lusi.

Mineral	MSWI BA	FA	Lusi
Akermanite	2.1	—	—
Quartz	5.3	4.6	8.0
Mullite	3.8	11.1	—
Calcite	2.3	—	0.4
Anhydrite	—	0.3	1.0
Magnetite	0.6	3.0	—
Wuestite	0.4	—	—
Sodalite	—	0.4	—
Iron	0.4	—	—
Cristobalite	0.1	—	—
MgAlSiO	0.7	—	—
Feldspar	2.8	—	16.3
Hematite	1.0	—	4.1
Vermiculite	—	—	1.7
Anatase	—	—	0.6
Corundum	1.9	—	—
Apatite	2.2	—	—
(Na,K)Cl	1.4	—	—
Periclase	1.4	—	—
Perovskite	—	0.3	—
Dolomite	0.9	—	—
Susannite	0.1	—	—
Amorphous	72.8	80.4	67.9

**Table 5:** Mass density and water absorption of the resulting LWA.

Test	Lusi 6 M	FA 6 M	MSWI BA 6 M	EC LWA
Apparent particle density ρ_a_ (kg/m^3^)	2.60 ± 0.09	2.23 ± 0.01	2.61 ± 0.03	1.25 ± 0.01
Oven Dried Density ρ_rd_ (kg/m^3^)	1.40 ± 0.02	1.47 ± 0.01	1.59 ± 0.01	0.99 ± 0.01
SSD particle density ρ_ssd_ (kg/m^3^)	1.86 ± 0.03	1.81 ± 0.00	1.98 ± 0.01	1.19 ± 0.01
Water absorption (%)	32.8 ± 0.28	23.69 ± 0.63	24.80 ± 0.56	21.14 ± 0.30
Porosity (%)	45.98 ± 0.96	34.12 ± 0.75	39.32 ± 0.78	20.85 ± 0.09

**Table 6:** Mineralogy of MSWI BA 6 M, FA 6 M, and Lusi 6 M LWA.

Mineral	MSWI BA 6 M	FA 6 M	Lusi 6 M
akermanite	1.8	—	—
quartz	3.9	4.0	5.7
mullite	—	6.9	—
calcite	5.0	0.7	0.4
magnetite	2.4	—	—
wuestite	0.3	—	—
AlFe3	—	0.2	—
silimanite	—	1.0	—
gupeiite	—	0.1	—
gaylusite	—	1.7	—
sodalite	—	0.2	—
magnesioferrite	—	2.2	—
iron	0.3	—	—
cristobalite	0.2	—	—
MgAlSiO	0.1	—	—
feldspar	1.8	—	10.8
hematite	0.3	1.2	2.6
vermiculite	—	—	1.0
ulvoespinel	—	—	1.3
anatase	—	—	0.5
corundum	0.9	—	—
apatite	0.3	—	—
(Na,K)Cl	0.7	—	—
tobermorite 11 a	3.7	—	—
perovskite	1.9	—	—
ankerite	0.9	—	—
amorphous	75.3	82.0	77.6
